# Methyl Internal Rotation in Fruit Esters: Chain-Length Effect Observed in the Microwave Spectrum of Methyl Hexanoate

**DOI:** 10.3390/molecules27092639

**Published:** 2022-04-20

**Authors:** Nhu-Ngoc Dang, Hoang-Nam Pham, Isabelle Kleiner, Martin Schwell, Jens-Uwe Grabow, Ha Vinh Lam Nguyen

**Affiliations:** 1 Department of Life Sciences, University of Science and Technology of Hanoi, Vietnam Academy of Science and Technology, 18 Hoang Quoc Viet, Hanoi, Vietnam; ngocdn.1999@gmail.com (N.-N.D.); pham-hoang.nam@usth.edu.vn (H.-N.P.); 2 Université Paris Cité and Univ Paris Est Creteil, CNRS, LISA, 75013 Paris, France; isabelle.kleiner@lisa.ipsl.fr; 3 Univ Paris Est Creteil and Université Paris Cité, CNRS, LISA, 94010 Créteil, France; martin.schwell@lisa.ipsl.fr; 4 Institut für Physikalische Chemie und Elektrochemie, Gottfried-Wilhelm-Leibniz-Universität Hannover, Callinstraße 3A, 30167 Hannover, Germany; 5 Institut Universitaire de France (IUF), 75231 Paris, France

**Keywords:** microwave spectroscopy, rotational spectroscopy, internal rotation, large amplitude motion

## Abstract

The gas-phase structures of the fruit ester methyl hexanoate, CH_3_-O-(C=O)-C_5_H_11_, have been determined using a combination of molecular jet Fourier-transform microwave spectroscopy and quantum chemistry. The microwave spectrum was measured in the frequency range of 3 to 23 GHz. Two conformers were assigned, one with C_s_ symmetry and the other with C_1_ symmetry where the γ-carbon atom of the hexyl chain is in a *gauche* orientation in relation to the carbonyl bond. Splittings of all rotational lines into doublets were observed due to internal rotation of the methoxy methyl group **CH_3_**-O, from which torsional barriers of 417 cm^−1^ and 415 cm^−1^, respectively, could be deduced. Rotational constants obtained from geometry optimizations at various levels of theory were compared to the experimental values, confirming the soft degree of freedom of the (C=O)-C bond observed for the C_1_ conformer of shorter methyl alkynoates like methyl butyrate and methyl valerate. Comparison of the barriers to methyl internal rotation of methyl hexanoate to those of other CH_3_-O-(C=O)-**R** molecules leads to the conclusion that though the barrier height is relatively constant at about 420 cm^−1^, it decreases in molecules with longer **R**.

## 1. Introduction

Methyl hexanoate (MHO) is a volatile compound in different kind of fruits such as pineapple fruit [1], passion fruit [2], acerola fruits [3], and strawberries [4], as identified by gas chromatography (GC). In more detail, the amount of MHO in pineapple fruit juice that is made from fresh cut is 0.015–3.8 mg/mL but it is significantly different in commercial pineapple juice products [1]. The peak area of MHO in strawberry is about 6.3 in newly harvested strawberry and it decreases after a few days of storage [4]. On the other hand, MHO is not very abundant in the other two types of fruits: the relative area of GC flame-ionization detection chromatogram is only 0.21% in organic yellow passion fruit [2] and a minor amount of it is detected in the intermediate state of maturity in acerola fruits [3]. Due to the effects of many factors like maturity state, storage condition, species and loss during processing, it is difficult to produce a typical juice with a fixed amount of ingredients and sensorial properties. The combination of many volatile compounds with different ratios produces the specific flavor and scent of the fruit [5]. Therefore, the gas-phase structures of methyl hexanoate might give important information towards a reasonable explanation for the structure–odor relation, as well as detect and quantify it in a mixture of various substances.

Furthermore, MHO can also be found at a large amount in corn oil after deep frying for 30 h [6]. The appearance of MHO in volatile decomposition products of deep fat frying shows that MHO can be used to produce deep fat fried flavor for non-fried food. Knowledge on the structures of this compound can pave the way to create expected flavoring. The physicochemical characteristics of volatile compounds is very essential in the acceptance of consumers as it mainly influences the olfactory system and combines with other conditions during the consumption of food like temperature, pressure, time, etc. The overall structure of food emulsion also depends on the composition and properties of each component that exists inside. MHO is one of those volatile compounds whose structures might be important to indicate their contribution to the final sensorial properties of food [7].

Microwave spectroscopy was established almost a century ago with a lot of interest since then [8]. The technique features many applications, two of them are (i) gas phase structure determination in terms of conformational analysis and (ii) large amplitude motion (LAM) in terms of methyl internal rotation [9]. (i) This applies mostly for small and medium-sized molecules, and therefore is ideal to study volatile compounds and to analyze their many different conformers. The gas phase structures of these compounds can serve further as inputs to research the odor–structure relation or create expected additives based on the olfactory mechanism and flavor preference of consumers [10,11,12]. Moreover, the results can also be used for benchmarking quantum chemical calculations. (ii) In addition to molecular structures, the effects of LAMs cause additional splittings in the microwave spectra, which need to be considered by appropriate quantum mechanical Hamiltonian models. Internal rotation, a kind of LAM, describes the rotation of a small group like a methyl group with respect to the rest of a molecule. Like the rotational constants for structure determination, the torsional barrier is a crucial parameter to illustrate internal rotation [13].

Microwave spectroscopy has been used to investigate many compounds belonging to the methyl alkanoate family, CH_3_-O-(C=O)-R, such as methyl acetate (R = CH_3_) [14,15], methyl propionate (R = C_2_H_5_) [16], methyl butyrate (R = C_3_H_7_) [17], and methyl valerate (R = C_4_H_9_) [18], leaving continued study of MHO (R = C_5_H_11_) to understand not only the conformational landscape but also the internal rotation of the methoxy methyl group **CH_3_**-O. In methyl butyrate and methyl valerate, where the alkyl chain is sufficiently long, two conformers were observed: one with a straight alkyl chain and a C_s_ symmetry, the other with a C_1_ symmetry, where the alkyl chain is bent at the γ-position of the chain [17,18]. For all molecules, the methoxy methyl group undergoes internal rotation with a torsional barrier starting from 424.6 cm^−1^ in methyl acetate, which then continously decreases over 422.8 cm^−1^ in methyl propionate and 420.2 cm^−1^ in methyl butyrate to 418.1 cm^−1^ in methyl valerate. In a study on linear aliphatic ketones, Andresen et al. reported on the so-called “chain length effect”, observing that the longer the alkyl chain, the lower the methyl torsional barrier, until a plateau is reached [19]. The continous decrease observed for methyl propionate, methyl butyrate, and methyl valerate strongly supports the trend, but suggests that the plateau is not yet reached at methyl valerate. Therefore, information obtained from MHO will yield an important data point to understand this effect in a further class of molecule other than the methyl *n*-alkyl ketones.

The present study thus aimed to determine the gas phase structure and obtain information on the methyl internal rotation of MHO by high resolution microwave spectroscopy with support from state-of-the-art calculations and computer codes for modelling and simulating the spectrum. 

## 2. Results

### 2.1. Quantum Chemical Calculations

**Figure 1:**
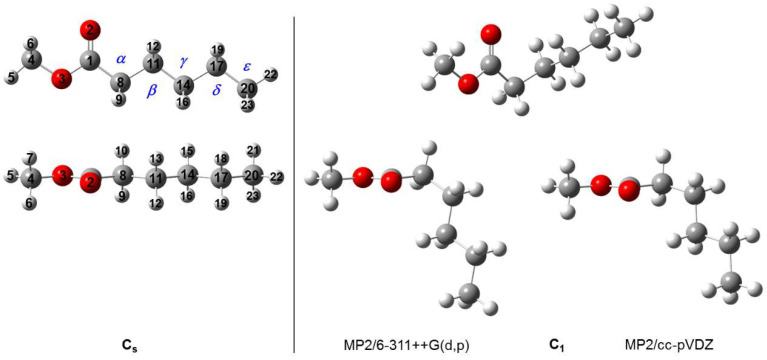
The two observed conformers C_s_ (**left** hand side) and C_1_ (**right** hand side) of MHO optimized at the MP2/6-311++G(d,p) and MP2/cc-pVDZ levels of theory. Atom numbering is given at the C_s_ conformer. Grey atoms are carbon, white atoms are hydrogen and the red ones are oxygen. Upper trace: View on the O–(CO)–C plane; lower trace: view along the O2=C1 bond.

Methyl hexanoate MHO is an organic compound known as fatty acid methyl ester that includes a linear aliphatic chain attached at the carbon atom of a COO group and a methyl group attached at the oxygen atom, as illustrated in Figure 1. The conformational analysis of MHO was performed with the *Gaussian* 16 program [20]. The Møller Plesset perturbation theory of second order MP2 method [21] and Pople’s basis set 6-311++G(d,p) [22] was selected to optimize the starting geometries. We chose this combination because of its reasonable calculation time/accuracy ratio, as shown in many previous studies such as those on nicotine [23], the prism, cage, and book isomers of water hexamers [24], 3,5-difluorobenzyl alcohol [25], and 2-methyl-1,3-dithiolane [26]. 

The starting geometries of MHO were created by adjusting the dihedral angles ϑ_1_ = ∡(O3−C1−C8−C11), ϑ_2_ = ∡(C1−C8−C11−C14), ϑ_3_ = ∡(C8−C11−C14−C17), and ϑ_4_ = ∡(C11−O14−C17−C20). The rotations about the O3−C4 and C17−C20 bonds correspond to the internal rotations of the methoxy **CH_3_**-O- and the alkyl methyl CH_2_-**CH_3_** groups, respectively, which do not produce new conformers. Varying the dihedral angle ϑ_5_ = ∡(C4−O3−C1−C8) leads to two stable conformations, *anti* with ϑ_5_ = 180° and *syn* with ϑ_5_ = 0°, where the former is known to be much more stable [27]. Due to the long alkyl chain of MHO, we only performed the conformation analysis based on the *anti* configurations with a starting value of ϑ_5_ = 180°. The adjustment of a dihedral angle ∡(A−B−C−D) is implemented by rotating the two planes A−B−C and B−C−D so that it always has the B−C axis in common. The ϑ*_i_* angles (*i* = 1–4) were set to 180°, ± 60°, and 0° in order to create 4^4^ = 256 starting geometries of MHO. Each geometry was optimized to the closest energy minimum. The geometry optimization results contain values of the rotational constants, dipole moment components, MP2 energies, and optimized dihedral angles for 36 conformers. Table 1 summarizes information of the two eventually assigned conformers. Images of 14 conformers with an energy cut-off at 4 kJ/mol as well as results for 25 conformers with a cut-off at 5 kJ/mol are in Figure S1 and Table S1 in the Supplementary Material, respectively. Subsequent frequency calculations confirmed all conformers to be true minima and not saddle points.

The conformer named conformer I with C_1_ symmetry is the most stable geometry and is considered to be most likely to appear in the spectrum under molecular beam conditions. We thus first focused on this conformer. Finally, we also assigned conformer XIII with C_s_ symmetry (see Section 2.2.). The optimized structures of these two conformers are illustrated in Figure 1. Their Cartesian coordinates are given in Table S2 in the Supplementary Material.

After the two conformers C_1_ and C_s_ of MHO were assigned, their geometries were also optimized using other methods and basis sets for benchmarking purposes. The methods in use consist of MP2 [21], B3LYP [28,29] with Grimme’s dispersion correction [30] with or without Becke–Johnson damping [31], and Truhlar’s method [32], each in combination with 16 basis sets [22,33]. The B3LYP method is also extended with the Coulomb-attenuating method (CAM) [34]. Such method/basis set combinations have been used popularly and yielded good results to various types of molecules [35,36,37,38]. Results from this basis set variation, mainly including the rotational constants and their differences to the experimental values, are summarized in Table S3.

Internal rotation of the methoxy methyl group in MHO is the main cause of doublet splittings (called the A-E splittings) observed for all rotational transitions in the microwave spectrum (see Section 2.2). The methyl group at the end of the alkyl chain, on the other hand, does not cause any additional splittings since the barrier to this methyl torsion is high. Though small splittings are still sometimes observed, such as for a series of aliphatic ketones [39,40,41] or in methyl propyl sulfide [42], this was not the case for all methyl alkynoates with a shorter alkyl chain than that of MHO. The barrier height of the methoxy methyl group of MHO is estimated to be very close to the value of about 420 cm^−1^ observed for methyl butyrate [17] and 418 cm^−1^ for methyl valerate [18]. Therefore, no quantum chemical calculations were performed to predict the barrier.

### 2.2. Microwave Spectroscopy

#### 2.2.1. Measurements

All measurements were recorded using a modified version of the molecular jet Fourier-transform microwave spectrometer described in Ref. [43], an instrument with an experimental accuracy of 2 kHz [44] operating in the frequency range from 2 to 26.5 GHz. The substance was purchased from TCI Europe, Zwijndrecht, Belgium, with a stated purity of 98%. Some drops of the sample were put on a piece of pipe cleaner inserted into a stainless-steel tube placed upstream of the nozzle. Helium was flown over the substance at a backing pressure of 2 bar. The helium-MHO mixture was expanded through a nozzle as a molecular beam into a chamber in which a vacuum of approximately 10^−7^ mbar was generated using an oil diffusion pump and a rotary pump. The molecular beam can propagate almost without collisions, resulting in a higher intensity and smaller width of the measured lines. Due to the expansion into the vacuum, the rotational temperature cools down to about 0.5–2 K because of the Joule–Thompson effect, so that only rotation levels of the vibrational ground state are occupied. Inside the vacuum tank is a Fabry–Perot resonator, consisting of two confocal mirrors of 80 cm diameter in a co-axial arrangement with the molecular beam (COBRA). With these two mirrors, an electric field in the form of a standing electromagnetic wave is created, whose frequency can be influenced by changing the position of a mirror. The COBRA arrangement increases the distance traveled by the molecular beam, therefore also increasing the line intensity and resolution. A macroscopic oscillating dipole moment is generated by the interaction of a microwave pulse whose frequency is close to a rotational transition of MHO. This polarization decays as soon as the microwave pulse is turned off, resulting in a microwave signal that is recorded in the time domain. The signal is digitized via an A/D converter and the microwave spectrum in the frequency domain is obtained using Fourier transformation.

**Figure 2:**
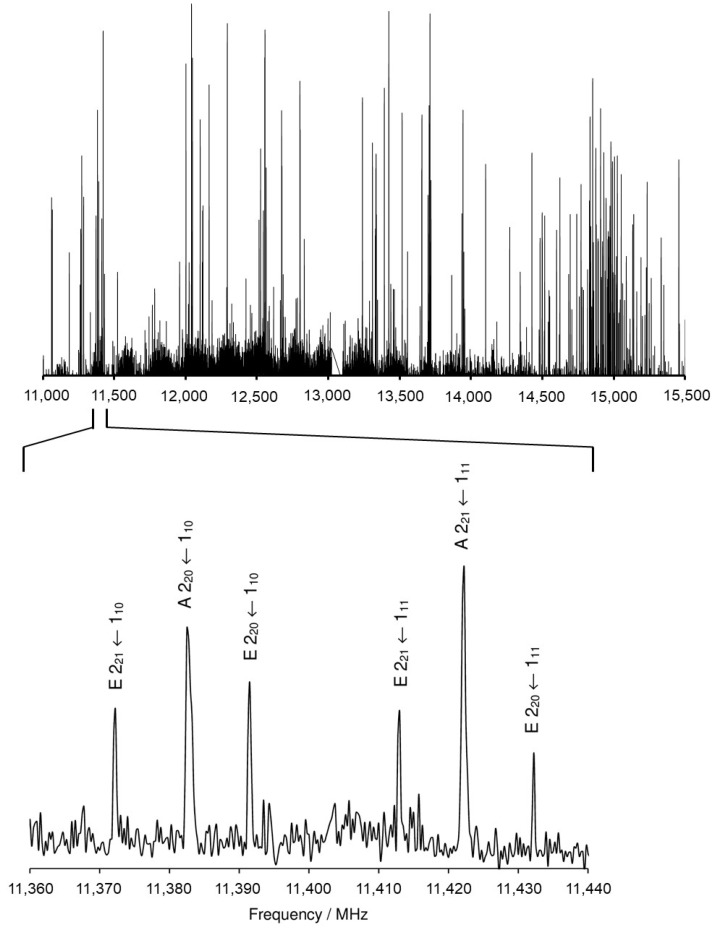
The survey spectrum of MHO recorded from 11,000 MHz to 15,500 MHz. A portion of the scan from 11,360 MHz to 11,440 MHz is illustrated in an enlarged scale where assigned lines are labeled with their corresponding quantum numbers *J*, *K_a_*, *K_c_* and torsional species (A or E). They all belong to conformer I. The intensities are in arbitrary units and a logarithmic scale.

**Figure 3:**
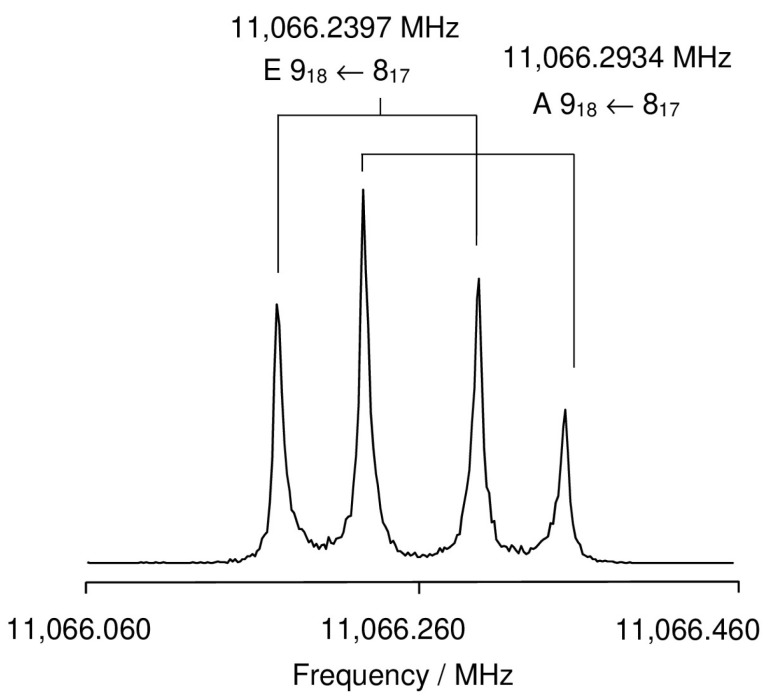
A high-resolution measurement of the 9_18_ ← 8_17_ transition of conformer I of MHO. The polarization frequency is 11,066 MHz for this spectrum, and 96 free induction decays were co-added.

At the beginning, we recorded a survey spectrum (scan) of MHO by taking overlapping spectra in the frequency range from 11.0 to 15.5 GHz with a step width of 0.25 MHz, as shown in Figure 2 with an enlarged portion in the range from 11,360 MHz to 11,440 MHz. The *XIAM* program [45] was used to predict a theoretical spectrum, which was then compared to the scan. When some lines in the scan had been assigned, measurements were performed directly at high-resolution between 3 and 22.3 MHz, where all signals appear as doublets due to the Doppler effect. The Doppler splitting approximately equals 0.001% of the value of the measured frequency under our molecular beam conditions. The arithmetic mean values of the Doppler peaks were taken as transition frequencies. The internal rotation of the methoxy methyl group caused another kind of splitting of all rotational transitions into an A species line and an E species line. Figure 3 shows an example of the 9_18_ ← 8_17_ transition where both types of splittings coincide in one high resolution spectrum. 

#### 2.2.2. The C_1_ Conformer

From our conclusion in Ref. [18] on methyl valerate, the rotational constants and dipole moment components of MHO calculated at the MP2/cc-pVDZ level (see Table 1) were input in *XIAM* to predict the microwave spectrum. Not only from the investigations on methyl valerate, but also from those of methyl butyrate, we observed the so-called “Θ-problem” for all C_1_ conformers. The soft degree of freedom around the C1-C8 bond makes the molecules extremely flexible and therefore, differences in the rotational constants calculated at different levels of theory are remarkable (up to 0.5 GHz), as can be seen in the benchmark on methyl valerate. For these conformers, the MP2/6-311++G(d,p) level completely fails to deliver equilibrium rotational constants which accidentally match the experimental constants. The MP2/cc-pVDZ level with its error compensations yielded an almost exact match between the predicted and the experimental rotational constants, which then made the assignments straightforward. Therefore, this level was used to aid the assignment of the microwave spectrum of MHO. First of all, we attempted to assign the A species transitions and ignored the internal rotation effects. By comparing the theoretical spectrum and the experimental survey spectrum, 12 lines were identified straightforwardly, among them 4 *a*-type, 3 *b*-type, and 5 *c*-type transitions. Fitting the line frequencies yielded new rotational constants, which were then used to predict the whole spectrum. At this point, high resolution spectra were recorded and we achieved a fit with 100 A species lines using the following Hamiltonian, which includes a rigid rotor Hamiltonian supplemented with quartic centrifugal distortion corrections:

(1) $$H_{\mathrm{rot}}=AP_{a}^{2}+BP_{b}^{2}+CP_{c}^{2}-\Delta_{J}P^{4}-\Delta_{JK}P^{2}P_{a}^{2}-\Delta_{K}P_{a}^{4}-2\delta_{J}P^{2}\left(P_{b}^{2}-P_{c}^{2}\right)-\delta_{K}\left\{P_{a}^{2},\left(P_{b}^{2}-P_{c}^{2}\right)\right\}$$

where *A, B, C* are the rotational constants, $P_{g}$ (*g* = *a*, *b*, *c*) the components of the total rotational angular momentum, $\Delta_{J}$, $\Delta_{JK}$, $\Delta_{K}$, $\delta_{J}$, and $\delta_{K}$ the quartic centrifugal distortion constants, and {u,v} denotes the anti-commutator uv + vu.

To analyze the internal rotation effects of the methoxy methyl group, the Hamiltonian was extended by a **H**_ir_ term:

(2) $$H_{\mathrm{ir}}=F{\left(p_{\alpha }-\rho_{a}P_{a}-\rho_{b}P_{b}-\rho_{c}P_{c}\right)}^{2}+V\left(\alpha \right)\;$$

*F* is the *reduced* internal rotation constant of the methyl group (which is related to the internal rotation constant *F*_0_ of the methyl group, see Equation (3)), $p_{\alpha }$ the internal rotation angular momentum conjugate to the torsional angle *α*, and $\rho_{g}$ are the components of the *ρ* vector. $\rho_{g}$ are related to the principal moments of inertia $I_{g}$ of the molecule and to the moment of inertia of the top $I_{\alpha }$ by the expressions:

(3) $$A=\frac{\hbar^{2}}{2I_{a}},\;B=\frac{\hbar^{2}}{2I_{b}},\;C=\frac{\hbar^{2}}{2I_{c}},\;F=\frac{F_{0}}{r}=\frac{\hbar^{2}}{2rI_{\alpha }},\;\rho_{g}=\frac{\lambda_{g}I_{\alpha }}{I_{g}},\;r=1-\underset{g}{\sum }\lambda_{g}^{2}\frac{I_{\alpha }}{I_{g}}$$

where $\lambda_{g}=\cos∠\left(i,g\right)$ are the direction cosines of the internal rotation axis *i* of the methyl internal rotor in the principal axis system.

Finally, the term V(α) accounts for the three-fold potential of the methyl top:

(4) $$V\left(\alpha \right)=\frac{1}{2}V_{3}\left(1-cos3\alpha \right)+\frac{1}{2}V_{6}\left(1-cos6\alpha \right)+\frac{1}{2}V_{9}\left(1-cos9\alpha \right)\ldots$$

Which was truncated after the first term. The $V_{3}$ potential in Equation (4), called the barrier height, of MHO was considered to be the same as that of methyl valerate which is about 418 cm^−1^ [18]. The angles between the internal rotor axis and the molecular principal axes were taken from ab initio and inserted in *XIAM* to predict the E species line frequencies. Since the experimental spectra were measured in the vibrational ground state, the methyl rotor constant is strongly correlated with the torsional barrier. We began the assignment with *a*-type transitions where A-E splittings are small and often visible in a high resolution measurement (see Figure 3 for example). The final fit, shown in Table 2, contains 180 fitted lines with a standard deviation of 4.0 kHz, which is the estimated measurement accuracy. The measurement accuracy was calculated as 1/10 of the average of all line widths at half maximum and is larger than the experimental accuracy, because many lines are blurred or broadened due to unresolved splittings. Some lines feature small additional splittings, probably due to internal rotation of the alkyl methyl group. We tried to refit the data set using the program *BELGI-C_1_* [46], which can include more higher order terms, but the deviation remains essentially the same. A list of all fitted transitions using the *XIAM* code along with their residues is given in Table S4 in the Supplementary Material. The *BELGI-C_1_* parameters are available in Table S5. 

#### 2.2.3. The C_s_ Conformer

After the assignment of the C_1_ conformer, there were less intense lines remaining unassigned in the scan, which may belong to other conformers of MHO. From the investigations on all methyl alkynoates with shorter alkyl chain, a conformer with a straight alkyl chain (called the C_s_ conformer) has always been observed. This is also the case for the alkyl acetate [47,48,49] as well as the methyl alkyl ketone series [19,39,40,41]. For MHO, this conformer (XIII) is predicted to be surprisingly high in energy, 3.25 kJ/mol higher than the global minimum conformer I. For conformers with C_s_ symmetry, the Θ-problem does not occur and the predicted rotational constants are relatively stable at different levels of theory, as shown in the benchmark on methyl valerate. In those cases, it is not critical which level of theory we use to start the assignment. Since for methyl valerate, the MP2/6-311++G(d,p) rotational constants are reasonable starting values to assign the C_s_ conformer [18], we also used the rotational constants predicted at this level for the initial assignment of the C_s_ conformer of MHO. Similar to the situation of methyl valerate, only the dipole moment component in the *b*-direction is sufficiently strong to be observed (see Table 1). In addition, only the *R*-branch *K_a_* = 1 ← 0 transitions are intense and within the frequency range measurable with the spectrometer. Therefore, the assignment was very challenging (three transitions are in the recorded survey spectrum). Finally, 44 lines, equally distributed between A and E species, could be measured and fitted to a standard deviation of 4.4 kHz. The program *BELGI-C_s_* [50] yielded a similar fit quality (see Table S5 in the Supplementary Material). Note that three E species *c*-type forbidden transitions are observed. For these transitions, the *K_a_* and *K_c_* quantum numbers have no meaning for the symmetry of the rotational transitions and only indicate the order of energy. The fitted parameters are also collected in Table 2. A list of all fitted frequencies along with their residuals are given in Table S6.

## 3. Discussion

We assigned two conformers in the microwave spectrum of MHO. The spectra were fitted using two programs *XIAM* and *BELGI* and were reproduced to measurement accuracy. The rotational constants and the torsional barrier of the methoxy methyl group were determined among other structural and internal rotation parameters. 

First, we will discuss the structural aspects of the conformers generally observed in methyl alkynoate. Independent on the length of the alkyl chain, one conformer is always observed in the microwave spectrum, featuring an all-*anti* geometry and therefore called the C_s_ conformer. The second type of conformer identified in the spectrum is the one with C_1_ symmetry and a configuration where the *β*-carbon is slightly tilted out of the C-(C=O)-C_α_ plane (ϑ_1_ ≈ 160°), while the *γ*-carbon is in a synclinal position (ϑ_2_ ≈ 70°) (for carbon labeling, see Figure 1). Methyl acetate and methyl propionate are not sufficiently long to support such a structure [14,15,16]. In the case of methyl valerate [18] and MHO, the supplementary *δ* and *ε*-carbon atoms are all in *anti* positions (ϑ*_i_*≈ 180°, with *i* = 3 and 4, respectively). Benchmark calculations performed for methyl butyrate and methyl valerate revealed the so-called Θ-problem, where the soft degree of freedom of the C1−C8 bond causes the entire alkyl chain to tilt out of the O(CO)C plane by an angle Θ = 180° − |ϑ_1_|. This leads to the observation that, while generally the benchmarking levels of theory yield rotational constants that differ by about 1% or less to the experimental values, we experience deviations of up to 15% for molecules with the Θ-problem. Benchmarkings on methyl butyrate and methyl valerate have shown that the MP2/cc-pVDZ level predicts most reliable rotational constants to start the assignment [17,18], which finally turned out to also be the case for MHO (see Table 1 and Table S3 of the Supplementary Material). We therefore recommend this level of theory for the assignment of the most stable conformer of longer methyl alkynoates. 

**Figure 4:**
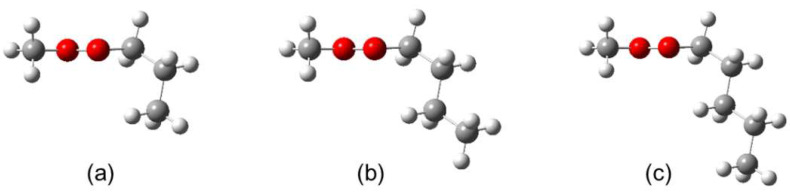
The most stable conformer I of (**a**) methyl butyrate, (**b**) methyl valerate, and (**c**) methyl hexanoate optimized at the MP2/6-311++G(d,p) level of theory. For atom colors, see Figure 1.

As shown in Figure 4, conformer I of methyl butyrate, which is the most stable one [17], is a sub-structure of the most stable conformer of methyl valerate [18]. An additional CH_2_ group is added to the alkyl chain of methyl butyrate in an antiperiplanar position to form methyl valerate. Similarly, conformer I of MHO also contains conformer I of methyl valerate as a sub-structure. Therefore, the most stable conformers of methyl alkynoates with longer alkyl chains, for example methyl heptanoate or methyl octanoate, can most probably be predicted by adding another CH_2_ group in an antiperiplanar position to the alkyl chain of conformer I of MHO. We note that the conformational analysis of MHO was performed while the assignment of methyl valerate was still in progress. Lacking benchmarking results, the conformational analysis was performed at the MP2/6-311++G(d,p) level of theory. For further investigations on methyl alkynoates with longer alkyl chains, we propose the use of only two calculations: MP2/cc-pVDZ for the C_1_ conformer and MP2/6-311++G(d,p) for the C_s_ conformer.

Secondly, unlike the case of methyl alkyl ketone where it was often not clear wheather the C_s_ or the C_1_ conformer is more stable [19,39,40,41], the spectra of the C_1_ conformers of methyl alkynoates clearly dominate those of the C_s_ conformers. Hernandez-Castillo et al. determined a population ratio of 59/41 for the so-called (g ±, a) conformer of methyl butyrate (equivalent to the C_1_ conformer of MHO) vs. the (a,a) conformer (equivalent to the C_s_ conformer) [17] from the chirped-pulse spectrum, and proposed that the C_1_ conformer is stabilized by two weak hydrogen bonds between the oxygen atom of the carbonyl group and (i) one hydrogen atom attached to the *γ*-carbon and (ii) one hydrogen atom attached to the *β*-carbon. This might also hold true for MHO. A quantitative statement is not attempted due to the lack of dipole moment measurements and the trust in intensity of the set up in use.

On the dependence between the methyl torsional barrier in methyl alkynoate on the molecular structure, the same observation is found as for the acetates, i.e., the length and the conformation of the alkyl chain do not significantly affect the methoxy methyl torsional barrier. This is not the case for methyl alkyl ketones where the C_s_ and the C_1_ conformers possess significantly different barriers [19,39,40,41]. The barriers to internal rotation of the methoxy methyl group of methyl alkynoates have been always around 420 cm^−1^, as also observed for conformer I of 3-mercaptopropionate (423.79(73) cm^−1^) [51]. For MHO, the torsional barriers of 415.2 cm^−1^ for the C_1_ conformer and 416.9 cm^−1^ for the C_s_ conformer do not provide an exception. A slight decrease in the barrier height is observed by the longer alkyl chain, as can be recognized in Table 3. This chain length effect has also been reported for a series of methyl alkyl ketones, where Andresen et al. found that beyond a certain molecule length, this effect is no longer significant and a plateau of the values is reached [19,39,40,41]. For methyl alkyl ketones, this limit seems to be at the *ζ*-carbon position, as the barrier height is almost unchanged in heptan-2-one and octan-2-one [19]. Regarding the values in Table 3, we believe that the plateau is not yet at MHO and that the barriers observed for methyl heptanoate will be slightly lower than those found for MHO. It should be noted that the scale of these chain length effects is rather small, and that the barrier height is quite sensitive to the set of parameters used in the fitting process. The same set of fitted parameters is essential for a reasonable comparison. For example, the internal rotation constant *F*_0_ of the methyl group directly influences the **H**_ir_ term of the Hamiltonian (see Equations (2) and (3)). It often takes values between 158 GHz and 160 GHz and is a fixed parameter in the fit. The reason is that *F*_0_ is highly correlated with the *V*_3_ potential because only rotational transitions in the vibrational ground state are observable under the jet-cooled measurement conditions. Fixing *F*_0_ to different values will lead to different values of *V*_3_ (see for example Figure 5 in Ref. [52]). Since, in the fits of methyl acetate [14], methyl butyrate [17], methyl valerate [18], and the fits of MHO shown in Table 2, *F*_0_ was fixed to 158 GHz, it is necessary to readjust the *F*_0_ value fixed to 160 GHz in the methyl propionate fit [16] to 158 GHz for a reasonable comparison. For this reason, though the value of methyl acetate fits well in this trend, we still mention that it has to be considered with caution because of the coupling between the acetyl methyl and the methoxy methyl group, which does not occur in all other molecules in the methyl alkynoate family. We also note that while for the ketones, the chain length detection is almost lost for the C_1_ conformer once the alkyl chain is bent [19], it can still be detected for both the C_1_ and C_s_ conformers of methyl alkynoate.

## 4. Conclusions

The spectrum of MHO was analyzed using microwave spectroscopy combined with quantum chemical calculations, revealing the most stable conformer with C_1_ symmetry and a less stable conformer with C_s_ symmetry. The MP2/6-311++G(d,p) level was used to optimized the starting geometries, was successful in aiding the assignment of the C_s_ conformer, but failed for the C_1_ conformer, for which the MP2/cc-pVDZ level was much more helpful. The most stable structure of MHO contains as sub-structures those of the most stable conformers of shorter-chain methyl alkynoates such as methyl butyrate and methyl valerate. The torsional barrier deduced for the methoxy methyl group of MHO confirms that generally for methyl alkynoate, the value is about 420 cm^−1^, and a molecule with longer alkyl chain features a lower barrier (chain length effect).

## Figures and Tables

**Table 1:** Rotational constants (in MHz), dipole moment components (in Debye), dihedral angles (in degree), and energies relative to that of the lowest energy conformer I (E = −424.5296788 Hartree) of the two observed conformers of MHO (I and XIII) calculated at the MP2/6-311++G(d,p) level of theory.

Conf.	*A*	*B*	*C*	*μ_a_*	*μ_b_*	*μ_c_*	ϑ_1_	ϑ_2_	ϑ_3_	ϑ_4_	*E*
I	3101.3	664.2	616.4	–0.37	0.33	–1.62	–146.54	66.52	179.98	179.95	0.00
I ^a^	3598.9	626.6	584.6	–0.83	0.58	–9.11	–166.18	69.11	179.53	179.90	
XIII	6990.7	483.4	459.9	0.07	–1.72	0.00	180.00	180.00	180.00	180.00	3.25
Exp. ^b^	3599.0	625.4	585.8								

^a^ Values calculated at the MP2/cc-pVDZ level of theory for the most stable conformer I (see text). ^b^ Experimental values obtained for conformer I. Note the agreement with the values calculated at the MP2/cc-pVDZ level.

**Table 2:** Molecular parameters of the two assigned conformers I (C_1_) and XIII (C_s_) of MHO obtained from a fit with the program *XIAM*.

Par. ^a^	Unit	I = C_1_	XIII = C_s_
*A*	MHz	3599.03841(24)	6987.3630(32)
*B*	MHz	625.35951(20)	483.652(15)
*C*	MHz	585.83319(20)	460.364(16)
Δ*_J_*	kHz	0.17529(66)	0.0393(91)
Δ*_JK_*	kHz	−4.7630(45)	
Δ*_K_*	kHz	45.278(21)	6.13(65)
*δ_J_*	kHz	0.01954(32)	0.0027(11)
*δ_K_*	kHz	−0.824(82)	27.8(76)
*V*_3_ ^b^	cm^−1^	415.15(13)	416.890(96)
∠(*i*,*a*)	°	134.280(79)	158.82(12)
∠(*i*,*b*)	°	133.80(40)	68.82(12)
∠(*i*,*c*)	°	79.46(95)	90.0 ^c^
*N*_A_ */N*_E_^d^		100/80	21/21
*σ* ^e^	kHz	4.0	4.4

^a^ All parameters refer to the principal axis system. Watson’s A reduction in I^r^ representation was used. The errors in parentheses are in the unit of the last significant digits. ^b^ The *V*_3_ parameter is correlated to the moment of inertia *F*_0_ of the methyl rotor. Therefore, *F*_0_ was fixed to 158 GHz in both fits, a value often found for methyl groups and also used in Refs. [17,18], corresponding to a moment of inertia of *I*_α_ = 3.2 uÅ^2^. ^c^ Fixed due to symmetry. ^d^ Number of the A (N_A_) and E species lines (N_E_). ^e^ Standard deviation of the fit.

**Table 3:** A collection of methyl alkynoates and their respective torsional barriers of the methoxy methyl group (in cm^−1^).

Molecule	C_s_ Conformer	C_1_ Conformer
Methyl acetate [14]	424.581(56)	
Methyl propionate [16]	422.801(22) ^a^	
Methyl butyrate [17]	420.155(71)	419.447(59)
Methyl valerate [18]	418.059(27)	417.724(70)
Methyl hexanoate ^b^	416.890(96)	415.15(13)

^a^ The data set was refitted with the moment of inertia *F*_0_ fixed to 158 GHz for a reasonable comparision with other molecules. Therefore, the value differs from that of 429.324(23) cm^−1^ given in Ref. [16] where *F*_0_ was fixed to 160 GHz. ^b^ This work.

## Data Availability

Data is contained within the article and Supplementary Material.

## Supplementary Materials

The following supporting information can be downloaded at: https://www.mdpi.com/article/10.3390/molecules27092639/s1, Figure S1: The 14 most stable conformers of MHO obtained at the MP2/6-311++G(d,p) level of theory; Table S1: Rotational constants, dipole moment components, dihedral angles, and energies relative to that of the lowest energy conformer I of 25 conformers of MHO obtained at the MP2/6-311++G(d,p) level with an energy cut-off at 5 kJ/mol.; Table S2: Geometry parameters in the principal axes of inertia of conformer I and conformer XIII of MHO; Table S3: Rotational constants of conformer I and conformer XIII calculated at different levels of theory and their deviations to the experimental values; Table S4: Observed frequencies of conformer I; Table S5: Molecular constants in the rho axis system obtained by fits using the program BELGI; Table S6: Observed frequencies of conformer XIII.

## Sample Availability

Samples of MHO purchased from TCI Europe, Zwijndrecht, Belgium, are available from the authors (H.V.L.N.).
