# Supplementary material for: Methyl Internal Rotation in Fruit Esters: Chain-Length Effect Observed in the Microwave Spectrum of Methyl Hexanoate
Source: Molecules. 2022 Apr 20;27(9):2639. doi: 10.3390/molecules27092639 (PMC9105109; doi:10.3390/molecules27092639)
Supplement: Supplementary file 1 [file molecules-27-02639-s001.zip › molecules-1685128-supplementary.pdf]

Supplementary Material

Methyl internal rotation in fruit esters: Chain-length effect observed in the microwave spectrum of methyl hexanoate

Nhu Ngoc Dang [1], Hoang Nam Pham [1], Isabelle Kleiner [2], Martin Schwell [3], Jens-Uwe Grabow [4,] and Ha Vinh Lam Nguyen [3,5,]**

[1] *Department of Life Sciences, University of Science and Technology of Hanoi, Vietnam Academy of Science and Technology, 18 Hoang Quoc Viet, Hanoi, Vietnam*

[2] *Université Paris Cité and Univ Paris Est Creteil, CNRS, LISA, 75013 Paris, France*

[3] *Univ Paris Est Creteil and Université Paris Cité, CNRS, LISA, 94010 Créteil, France*

[4] *Institut für Physikalische Chemie und Elektrochemie, Gottfried-Wilhelm-Leibniz-Universität Hannover, Callinstraße 3A, 30167 Hannover, Germany*

[5] *Institut Universitaire de France (IUF), 75231 Paris, France*

* *Correspondence: jens-uwe.grabow@pci.uni-hannover.de and lam.nguyen@lisa.ipsl.fr*

Figure S-1. The 14 most stable conformers of MHO obtained at the MP2/6-311++G(d,p) level of theory.

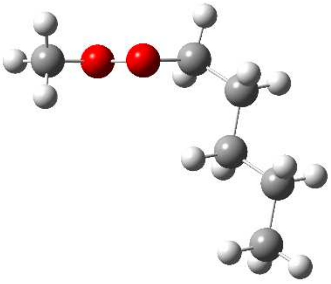

Conformer I

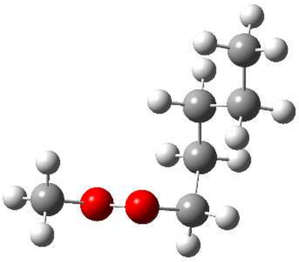

Conformer II

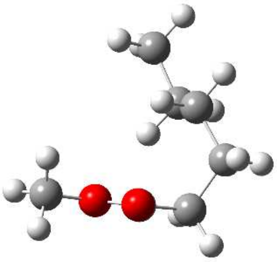

Conformer III

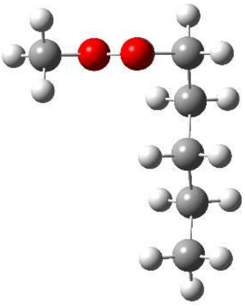

Conformer IV

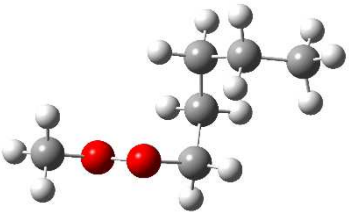

Conformer V

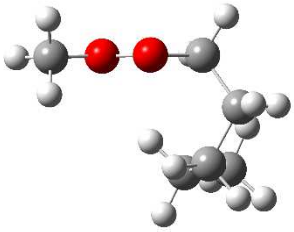

Conformer VI

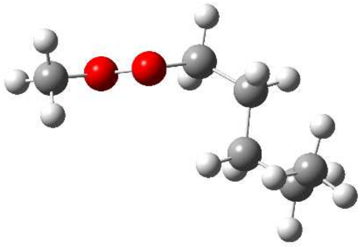

Conformer VII

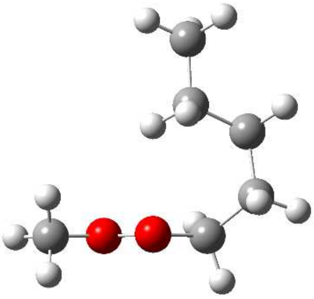

Conformer VIII

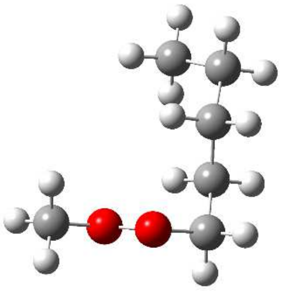

Conformer IX

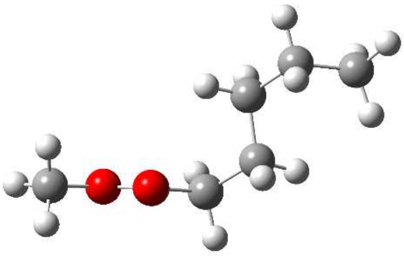

Conformer X

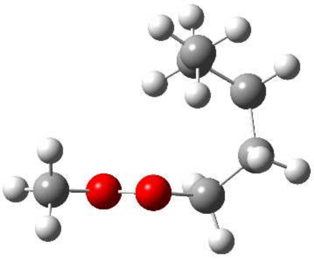

Conformer XI

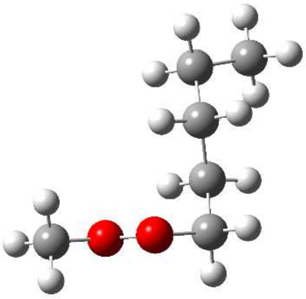

Conformer XII

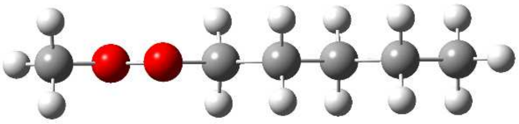

Conformer XIII

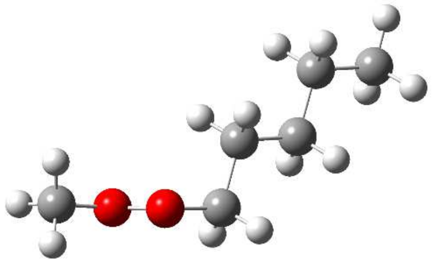

Conformer XIV

Table S-1. Rotational constants (in GHz), dipole moment components (in Debye), dihedral angles (in degress), and energies relative to that of the lowest energy conformer I (E = –424.5296788 Hartree) of 25 conformers of MHO obtained at the MP2/6-311++G(d,p) level of theory with an energy cut-off at 5 kJ/mol.

Conf.	A	B	C	μ_a	μ_b	μ_c	ϑ_1	ϑ_2	ϑ_3	ϑ_4	E
1	3101.3	664.2	616.4	–0.37	0.33	–1.62	–146.54	66.52	179.98	179.95	0.00
2	2533.0	867.0	793.1	–0.33	–1.38	–1.40	64.85	52.86	59.20	175.13	0.05
3	2142.4	1010.1	790.5	–1.08	0.91	1.33	113.48	–57.71	–60.27	–175.71	0.07
4	2548.4	748.8	691.4	0.70	–0.06	–2.07	–57.48	–57.45	–177.92	–179.22	0.74
5	3159.0	833.2	799.8	0.13	1.92	–0.45	63.23	53.71	56.28	57.98	1.09
6	2614.4	921.0	802.2	–0.85	–1.47	0.83	–120.81	58.82	56.34	57.94	1.13
7	3341.2	695.7	662.8	–0.55	1.28	–1.02	–144.95	66.90	176.74	63.39	1.67
8	2149.9	997.5	797.2	0.01	–0.18	–1.67	141.46	–79.74	59.80	174.62	2.06
9	2510.6	836.2	734.9	1.12	–0.97	1.54	58.23	58.34	175.31	63.15	2.22
10	4206.1	629.8	610.9	–0.34	0.85	1.40	147.98	–67.60	174.07	62.01	2.24
11	2219.7	1091.5	922.5	–0.27	0.61	1.62	137.60	–80.52	64.57	65.42	2.32
12	3142.2	720.1	674.5	0.94	0.97	–1.70	56.36	58.38	–176.77	–62.90	3.11
13	6990.7	483.4	459.9	0.07	–1.72	0.00	180.00	180.00	180.00	180.00	3.25
14	4745.5	519.6	500.5	–0.87	1.71	0.89	86.46	178.77	–179.79	179.94	3.26
15	2061.0	1027.6	859.5	0.43	0.40	–2.07	–49.41	– 70.53	67.61	173.81	4.09
16	1989.6	1258.0	980.4	0.86	–0.50	–1.85	–51.87	– 69.31	71.33	173.81	4.20
17	3090.0	734.3	642.0	1.34	–1.72	–0.16	82.16	175.58	58.21	56.90	4.40
18	3894.4	667.6	611.3	–0.29	–2.03	0.20	– 90.15	177.67	57.98	56.99	4.61
19	4969.4	544.2	513.2	0.30	–1.68	0.55	–167.74	176.04	62.93	173.77	4.66
20	5129.8	550.1	512.0	–0.27	–1.74	0.19	164.28	177.30	174.67	62.30	4.68
21	4277.3	576.6	544.6	1.11	–1.86	0.31	–81.94	–174.38	–62.56	–174.00	4.71
22	3816.2	623.1	621.2	–0.15	–0.09	1.80	161.29	–177.25	–58.70	–57.08	4.75
23	5085.2	554.7	529.3	–0.57	–1.89	–0.54	88.37	–176.57	–62.27	–174.05	4.86
24	3368.3	617.1	569.0	1.01	1.28	1.33	–87.38	–179.43	174.83	62.55	4.91
25	3589.3	584.1	580.7	0.72	–0.64	–1.92	87.47	178.13	174.95	62.44	4.93

Table S-2a. Geometry parameters in the principal axes of inertia of conformer I (C_1) and conformer XIII (C_s) of MHO calculated at the MP2/6-311++G(d,p) level of theory. The atoms are numbered according to Figure 1.

	**Conformer I**			**Conformer XIII**		
	***a* /Å**	***b* /Å**	***c* /Å**	***a* /Å**	***b* /Å**	***c* /Å**
C1	−1.440017	0.271433	0.170154	−1.793418	0.120542	−0.000002
O2	−1.349420	−0.127303	1.312377	−1.942101	1.323858	0.000012
O3	−2.355283	−0.193967	−0.710353	−2.824248	−0.757881	−0.000008
C4	−3.238542	−1.191265	−0.171502	−4.123607	−0.146083	−0.000003
H5	−3.908161	−1.454758	−0.988029	−4.831841	−0.972573	−0.000015
H6	−2.671713	−2.063446	0.158975	−4.254410	0.472937	0.889419
H7	−3.799877	−0.787740	0.673085	−4.254406	0.472959	−0.889410
C8	−0.583281	1.354522	−0.444403	−0.469999	−0.608490	0.000008
H9	−0.598886	1.250842	−1.533263	−0.450793	−1.267327	0.876450
H10	−1.065686	2.309626	−0.200924	−0.450796	−1.267360	−0.876409
C11	0.844808	1.320921	0.101071	0.727660	0.335591	−0.000010
H12	0.810463	1.384076	1.194256	0.672346	0.990072	0.877324
H13	1.383809	2.204296	−0.262526	0.672348	0.990038	−0.877369
C14	1.600719	0.058574	−0.314318	2.056707	−0.418990	0.000005
H15	1.625125	−0.004444	−1.411585	2.104531	−1.074485	−0.880860
H16	1.057172	−0.825312	0.044690	2.104530	−1.074452	0.880895
C17	3.030452	0.014011	0.224978	3.270855	0.510277	−0.000011
H18	3.575577	0.898719	−0.128325	3.220118	1.164009	−0.879766
H19	2.999453	0.079229	1.319741	3.220116	1.164041	0.879721
C20	3.775370	−1.253276	−0.196073	4.595374	−0.253753	0.000004
H21	3.834264	−1.323039	−1.287328	4.673103	−0.893921	−0.884959
H22	4.795566	−1.269348	0.199008	5.451423	0.427567	−0.000007
H23	3.256830	−2.145399	0.170082	4.673101	−0.893889	0.884992

Table S-2b. Geometry parameters in the principal axes of inertia of conformer I (C_1) and conformer XIII (C_s) of MHO calculated at the MP2/cc-pVDZ level of theory. The atoms are numbered according to Figure 1.

	**Conformer I**			**Conformer XIII**		
	***a* /Å**	***b* /Å**	***c* /Å**	***a* /Å**	***b* /Å**	***c* /Å**
C1	–1.436275	0.146521	0.161188	1.700610	0.103457	0.000391
O2	–1.185656	–0.659136	1.037927	1.840015	1.311577	0.000017
O3	–2.566262	0.110624	–0.588771	2.737282	–0.773260	–0.000001
C4	–3.456202	–0.962861	–0.241342	4.027704	–0.141692	–0.000408
H5	–4.311771	–0.868571	–0.921934	4.757244	–0.961389	–0.000696
H6	–2.962693	–1.937741	–0.374582	4.155084	0.487985	–0.894353
H7	–3.784731	–0.875530	0.805720	4.155684	0.487895	0.893515
C8	–0.573065	1.329560	–0.229600	0.378884	–0.635331	0.000254
H9	–0.571233	1.411385	–1.330159	0.365645	–1.302619	–0.880452
H10	–1.087669	2.233616	0.144282	0.365498	–1.302610	0.880963
C11	0.844640	1.230265	0.334373	–0.821048	0.308473	0.000142
H12	0.782979	1.056642	1.423184	–0.760153	0.971228	–0.881595
H13	1.357746	2.197870	0.186257	–0.760314	0.971226	0.881891
C14	1.669966	0.113718	–0.311049	–2.156053	–0.438590	0.000020
H15	1.717809	0.280132	–1.405614	–2.208262	–1.101740	0.885781
H16	1.158341	–0.853383	–0.154830	–2.208103	–1.101734	–0.885754
C17	3.092403	0.024345	0.247593	–3.366353	0.498973	–0.000085
H18	3.604724	0.993987	0.099805	–3.311119	1.160354	0.884829
H19	3.040271	–0.137074	1.340609	–3.310962	1.160359	–0.884984
C20	3.910454	–1.095055	–0.400246	–4.698575	–0.253944	–0.000205
H21	3.998210	–0.938563	–1.489352	–4.785466	–0.899699	0.890639
H22	4.929795	–1.147249	0.016827	–5.556310	0.438738	–0.000279
H23	3.428376	–2.074850	–0.240557	–4.785308	–0.899694	–0.891068

Table S-3a. The rotational constants *A*, *B*, *C* (in MHz) of conformer I of MHO calculated at different levels of theory and their deviations to the experimental values (calc.–exp.) Δ*A*, Δ*B*, and Δ*C*, respectively (in MHz).

Level	*A*	Δ*A*	*B*	Δ*B*	*C*	Δ*C*
B3LYP-D3/6-311G(3df,3pd)	3747.1	148.1	617.8	–7.6	577.3	–8.5
B3LYP-D3/6-311+G(3df,3pd)	3591.1	–7.9	622.7	–2.7	583.6	–2.2
B3LYP-D3/6-311++G(3df,3pd)	3588.1	–10.9	622.8	–2.6	583.8	–2.0
B3LYP-D3/6-311G(2d,2p)	3751.7	152.7	617.1	–8.3	576.3	–9.5
B3LYP-D3/6-311+G(2d,2p)	3584.4	–14.6	621.7	–3.7	582.7	–3.1
B3LYP-D3/6-311++G(2d,2p)	3580.5	–18.5	622.0	–3.4	583.0	–2.8
B3LYP-D3/6-311G(2df,2pd)	3776.9	177.9	616.7	–8.7	576.0	–9.8
B3LYP-D3/6-311+G(2df,2pd)	3602.1	3.1	621.6	–3.8	582.7	–3.1
B3LYP-D3/6-311++G(2df,2pd)	3596.7	–2.3	621.9	–3.5	583.0	–2.8
B3LYP-D3/6-311G(df,pd)	3748.7	149.7	617.4	–8.0	576.4	–9.4
B3LYP-D3/6-311+G(df,pd)	3556.7	–42.3	623.1	–2.3	584.1	–1.7
B3LYP-D3/6-311++G(df,pd)	3549.5	–49.5	623.6	–1.8	584.5	–1.3
B3LYP-D3/6-311G(d,p)	3749.2	150.2	615.1	–10.3	574.2	–11.6
B3LYP-D3/6-311+G(d,p)	3555.1	–43.9	621.0	–4.4	582.1	–3.7
B3LYP-D3/6-311++G(d,p)	3552.6	–46.4	621.2	–4.2	582.2	–3.6
B3LYP-D3/6-31G(d,p)	3658.4	59.4	620.1	–5.3	578.5	–7.3
B3LYP-D3/6-31+G(d,p)	3508.5	–90.5	621.3	–4.1	582.4	–3.4
B3LYP-D3/6-31++G(d,p)	3508.6	–90.4	621.3	–4.1	582.4	–3.4
B3LYP-D3/cc-pVDZ	3762.4	163.4	614.9	–10.5	573.1	–12.7
B3LYP-D3/cc-pVTZ	3687.4	88.4	618.3	–7.1	579.0	–6.8
B3LYP-D3/aug-cc-pVDZ	3568.7	–30.3	620.0	–5.4	580.9	–4.9
B3LYP-D3/aug-cc-pVTZ	3598.6	–0.4	621.5	–3.9	582.7	–3.1
B3LYP-D3BJ/6-311G(3df,3pd)	3691.3	92.3	620.5	–4.9	581.0	–4.8
B3LYP-D3BJ/6-311+G(3df,3pd)	3499.6	–99.4	627.7	2.3	589.2	3.4
B3LYP-D3BJ/6-311++G(3df,3pd)	3496.0	–103.0	627.9	2.5	589.4	3.6
B3LYP-D3BJ/6-311G(2d,2p)	3699.7	100.7	619.7	–5.7	579.8	–6.0
B3LYP-D3BJ/6-311+G(2d,2p)	3500.2	–98.8	626.5	1.1	588.0	2.2
B3LYP-D3BJ/6-311++G(2d,2p)	3494.6	–104.4	626.8	1.4	588.3	2.5
B3LYP-D3BJ/6-311G(2df,2pd)	3730.9	131.9	618.9	–6.5	579.1	–6.7
B3LYP-D3BJ/6-311+G(2df,2pd)	3515.5	–83.5	626.4	1.0	588.0	2.2
B3LYP-D3BJ/6-311++G(2df,2pd)	3510.8	–88.2	626.7	1.3	588.3	2.5
B3LYP-D3BJ/6-311G(df,pd)	3685.0	86.0	620.3	–5.1	580.4	–5.4
B3LYP-D3BJ/6-311+G(df,pd)	3462.0	–137.0	628.5	3.1	589.9	4.1
B3LYP-D3BJ/6-311++G(df,pd)	3454.7	–144.3	629.1	3.7	590.3	4.5
B3LYP-D3BJ/6-311G(d,p)	3687.9	88.9	617.9	–7.5	578.1	–7.7
B3LYP-D3BJ/6-311+G(d,p)	3461.8	–137.2	626.1	0.7	587.7	1.9
B3LYP-D3BJ/6-311++G(d,p)	3461.6	–137.4	626.2	0.8	587.7	1.9
B3LYP-D3BJ/6-31G(d,p)	3588.6	–10.4	623.8	–1.6	582.9	–2.9
B3LYP-D3BJ/6-31+G(d,p)	3404.1	–194.9	627.4	2.0	588.7	2.9
B3LYP-D3BJ/6-31++G(d,p)	3402.0	–197.0	627.5	2.1	588.8	3.0
B3LYP-D3BJ/aug-cc-pVDZ	3483.6	–115.4	624.7	–0.7	586.2	0.4
B3LYP-D3BJ/aug-cc-pVTZ	3508.2	–90.8	626.4	1.0	588.2	2.4
B3LYP-D3BJ/cc-pVDZ	3711.7	112.7	617.3	–8.1	576.5	–9.3
B3LYP-D3BJ/cc-pVTZ	3611.4	12.4	622.0	–3.4	583.6	–2.2
CAM-B3LYP-D3BJ/6-311G(d,p)	3780.0	181.0	620.7	–4.7	580.0	–5.8
CAM-B3LYP-D3BJ/6-311+G(d,p)	3590.7	–8.3	626.3	0.9	587.5	1.7
CAM-B3LYP-D3BJ/6-311++G(d,p)	3587.4	–11.6	626.5	1.1	587.7	1.9
CAM-B3LYP-D3BJ/aug-cc-pVDZ	3600.6	1.6	625.3	–0.1	586.4	0.6
CAM-B3LYP-D3BJ/aug-cc-pVTZ	3620.7	21.7	627.6	2.2	588.9	3.1

CAM-B3LYP-D3BJ/cc-pVDZ	3793.9	194.9	620.6	–4.8	578.7	–7.1
CAM-B3LYP-D3BJ/cc-pVTZ	3716.4	117.4	623.9	–1.5	584.8	–1.0
M06-2X/6-311G(3df,3pd)	3776.9	177.9	626.9	1.5	584.6	–1.2
M06-2X/6-311+G(3df,3pd)	3695.5	96.5	629.2	3.8	587.7	1.9
M06-2X/6-311++G(3df,3pd)	3691.7	92.7	629.4	4.0	587.9	2.1
M06-2X/6-311G(df,pd)	3772.9	173.9	626.5	1.1	584.0	–1.8
M06-2X/6-311+G(df,pd)	3659.4	60.4	630.0	4.6	588.5	2.7
M06-2X/6-311++G(df,pd)	3647.5	48.5	630.7	5.3	589.2	3.4
M06-2X/6-311G(d,p)	3773.9	174.9	624.7	–0.7	582.3	–3.5
M06-2X/6-311+G(d,p)	3660.8	61.8	628.1	2.7	586.8	1.0
M06-2X/6-311++G(d,p)	3653.5	54.5	628.5	3.1	587.2	1.4
M06-2X/6-31G(d,p)	3716.0	117.0	628.4	3.0	585.1	–0.7
M06-2X/6-31+G(d,p)	3641.5	42.5	627.7	2.3	586.2	0.4
M06-2X/6-31++G(d,p)	3639.5	40.5	627.8	2.4	586.3	0.5
M06-2X-aug-cc-pVDZ	3664.9	65.9	628.2	2.8	586.5	0.7
M06-2X-aug-cc-pVTZ	3691.6	92.6	628.7	3.3	587.6	1.8
M06-2X-cc-pVDZ	3778.3	179.3	625.6	0.2	582.1	–3.7
M06-2X-cc-pVTZ	3744.4	145.4	626.7	1.3	585.3	–0.5
MP2/6-311G(3df,3pd)	3590.8	–8.2	639.0	13.6	595.7	9.9
MP2/6-311G(2d,2p)	3620.2	21.2	634.0	8.6	591.1	5.3
MP2/6-311+G(2d,2p)	3361.9	–237.1	646.7	21.3	603.7	17.9
MP2/6-311++G(2d,2p)	3353.2	–245.8	647.2	21.8	604.1	18.3
MP2/6-311G(2df,2pd)	3659.8	60.8	636.1	10.7	592.9	7.1
MP2/6-311+G(2df,2pd)	3397.5	–201.5	648.5	23.1	605.4	19.6
MP2/6-311++G(2df,2pd)	3387.9	–211.1	649.0	23.6	605.9	20.1
MP2/6-311G(df,pd)	3458.9	–140.1	644.4	19.0	601.7	15.9
MP2/6-311+G(df,pd)	3120.6	–478.4	669.4	44.0	621.0	35.2
MP2/6-311++G(df,pd)	3117.7	–481.3	669.7	44.3	621.1	35.3
MP2/6-311G(d,p)	3445.3	–153.7	638.7	13.3	596.7	10.9
MP2/6-311+G(d,p)	3105.8	–493.2	663.7	38.3	616.1	30.3
MP2/6-311++G(d,p)	***3101.3***	***–497.7***	***664.2***	***38.8***	***616.4***	***30.6***
MP2/6-31G(d,p)	3524.1	–74.9	635.0	9.6	592.7	6.9
MP2/6-31+G(d,p)	3135.3	–463.7	659.0	33.6	613.0	27.2
MP2/6-31++G(d,p)	3050.4	–548.6	666.9	41.5	618.3	32.5
MP2/aug-cc-pVDZ	3095.3	–503.7	661.3	35.9	613.1	27.3
**MP2/cc-pVDZ**	**3598.9**	**–0.1**	**626.6**	**1.2**	**584.6**	**–1.2**
MP2/cc-pVTZ	3503.5	–95.5	640.5	15.1	598.5	12.7
ωB97X-D/6-311G(d,p)	3552.3	–46.7	625.6	0.2	587.5	1.7
ωB97X-D/6-311+G(d,p)	3549.4	–49.6	625.8	0.4	587.7	1.9
ωB97X-D/6-311++G(d,p)	3730.9	131.9	619.7	–5.7	580.2	–5.6
ωB97X-D/aug-cc-pVDZ	3573.1	–25.9	623.5	–1.9	585.4	–0.4
ωB97X-D/aug-cc-pVTZ	3597.1	–1.9	625.2	–0.2	587.7	1.9
ωB97X-D/cc-pVDZ	3749.1	150.1	618.1	–7.3	578.0	–7.8
ωB97X-D/cc-pVTZ	3685.0	86.0	621.4	–4.0	583.6	–2.2
**Expt.**	**3599.0**		**625.4**		**585.8**	

Table S-3b. The rotational constants *A*, *B*, *C* (in MHz) of conformer XIII of MHO calculated at different levels of theory and their deviations to the experimental values (calc.–exp.) Δ*A*, Δ*B*, and Δ*C*, respectively (in MHz).

**Level**	***A***	**Δ*A***	***B***	**Δ*B***	***C***	**Δ*C***
B3LYP-D3/6-311G(3df,3pd)	7057.8	70.4	482.2	–1.5	458.9	–1.5
B3LYP-D3/6-311+G(3df,3pd)	7047.3	59.9	481.4	–2.3	458.2	–2.2
B3LYP-D3/6-311++G(3df,3pd)	7047.4	60.0	481.4	–2.3	458.2	–2.2
B3LYP-D3/6-311G(2d,2p)	7056.6	69.2	481.6	–2.1	458.4	–2.0
B3LYP-D3/6-311+G(2d,2p)	7037.7	50.3	480.8	–2.9	457.6	–2.8
B3LYP-D3/6-311++G(2d,2p)	7037.7	50.3	480.8	–2.9	457.6	–2.8
B3LYP-D3/6-311G(2df,2pd)	7060.1	72.7	482.1	–1.6	458.9	–1.5
B3LYP-D3/6-311+G(2df,2pd)	7042.6	55.2	481.3	–2.4	458.1	–2.3
B3LYP-D3/6-311++G(2df,2pd)	7042.7	55.3	481.3	–2.4	458.1	–2.3
B3LYP-D3/6-311G(df,pd)	7040.5	53.1	481.5	–2.2	458.2	–2.2
B3LYP-D3/6-311+G(df,pd)	7026.0	38.6	480.7	–3.0	457.5	–2.9
B3LYP-D3/6-311++G(df,pd)	7026.1	38.7	480.7	–3.0	457.5	–2.9
B3LYP-D3/6-311G(d,p)	7022.0	34.6	480.1	–3.6	457.0	–3.4
B3LYP-D3/6-311+G(d,p)	7005.7	18.3	479.4	–4.3	456.2	–4.2
B3LYP-D3/6-311++G(d,p)	7005.7	18.3	479.4	–4.3	456.2	–4.2
B3LYP-D3/6-31G(d,p)	6996.4	9.0	480.1	–3.6	456.9	–3.5
B3LYP-D3/6-31+G(d,p)	6974.1	–13.3	478.4	–5.3	455.2	–5.2
B3LYP-D3/6-31++G(d,p)	6973.9	–13.5	478.4	–5.3	455.2	–5.2
B3LYP-D3/cc-pVDZ	6983.7	–3.7	480.2	–3.5	457.0	–3.4
B3LYP-D3/cc-pVTZ	7039.5	52.1	481.6	–2.1	458.4	–2.0
B3LYP-D3/aug-cc-pVDZ	6972.7	–14.7	479.3	–4.4	456.1	–4.3
B3LYP-D3/aug-cc-pVTZ	7035.7	48.3	481.3	–2.4	458.0	–2.4
B3LYP-D3BJ/6-311G(3df,3pd)	7065.6	78.2	482.9	–0.8	459.7	–0.7
B3LYP-D3BJ/6-311+G(3df,3pd)	7054.3	66.9	482.2	–1.5	459.0	–1.4
B3LYP-D3BJ/6-311++G(3df,3pd)	7054.4	67.0	482.2	–1.5	459.0	–1.4
B3LYP-D3BJ/6-311G(2d,2p)	7064.6	77.2	482.4	–1.3	459.2	–1.2
B3LYP-D3BJ/6-311+G(2d,2p)	7045.0	57.6	481.6	–2.1	458.3	–2.1
B3LYP-D3BJ/6-311++G(2d,2p)	7044.9	57.5	481.6	–2.1	458.3	–2.1
B3LYP-D3BJ/6-311G(2df,2pd)	7067.9	80.5	482.9	–0.8	459.7	–0.7
B3LYP-D3BJ/6-311+G(2df,2pd)	7049.6	62.2	482.1	–1.6	458.8	–1.6
B3LYP-D3BJ/6-311++G(2df,2pd)	7049.7	62.3	482.1	–1.6	458.8	–1.6
B3LYP-D3BJ/6-311G(df,pd)	7048.2	60.8	482.2	–1.5	459.0	–1.4
B3LYP-D3BJ/6-311+G(df,pd)	7033.0	45.6	481.4	–2.3	458.2	–2.2
B3LYP-D3BJ/6-311++G(df,pd)	7033.0	45.6	481.4	–2.3	458.2	–2.2
B3LYP-D3BJ/6-311G(d,p)	7029.5	42.1	480.9	–2.8	457.7	–2.7
B3LYP-D3BJ/6-311+G(d,p)	7012.7	25.3	480.1	–3.6	457.0	–3.4
B3LYP-D3BJ/6-311++G(d,p)	7012.7	25.3	480.1	–3.6	457.0	–3.4
B3LYP-D3BJ/6-31G(d,p)	7004.6	17.2	480.9	–2.8	457.6	–2.8
B3LYP-D3BJ/6-31+G(d,p)	6981.5	–5.9	479.2	–4.5	456.0	–4.4
B3LYP-D3BJ/6-31++G(d,p)	6981.2	–6.2	479.2	–4.5	456.0	–4.4
B3LYP-D3BJ/aug-cc-pVDZ	6980.0	–7.4	480.0	–3.7	456.8	–3.6
B3LYP-D3BJ/aug-cc-pVTZ	7042.6	55.2	482.1	–1.6	458.8	–1.6
B3LYP-D3BJ/cc-pVDZ	6991.3	3.9	480.9	–2.8	457.7	–2.7
B3LYP-D3BJ/cc-pVTZ	7046.6	59.2	482.4	–1.3	459.1	–1.3
CAM-B3LYP-D3BJ/6-311G(d,p)	7094.9	107.5	484.9	1.2	461.6	1.2
CAM-B3LYP-D3BJ/6-311+G(d,p)	7078.7	91.3	484.1	0.4	460.8	0.4
CAM-B3LYP-D3BJ/6-311++G(d,p)	7078.5	91.1	484.1	0.4	460.8	0.4
CAM-B3LYP-D3BJ/aug-cc-pVDZ	7049.4	62.0	483.9	0.2	460.6	0.2
CAM-B3LYP-D3BJ/aug-cc-pVTZ	7107.8	120.4	486.1	2.4	462.7	2.3

CAM-B3LYP-D3BJ/cc-pVDZ	7059.4	72.0	484.8	1.1	461.5	1.1
CAM-B3LYP-D3BJ/cc-pVTZ	7111.7	124.3	486.5	2.8	463.1	2.7
M06-2X/6-311G(3df,3pd)	7130.0	142.6	487.3	3.6	463.9	3.5
M06-2X/6-311+G(3df,3pd)	7121.6	134.2	486.7	3.0	463.3	2.9
M06-2X/6-311++G(3df,3pd)	7121.6	134.2	486.7	3.0	463.3	2.9
M06-2X/6-311G(df,pd)	7110.2	122.8	486.6	2.9	463.2	2.8
M06-2X/6-311+G(df,pd)	7099.0	111.6	486.0	2.3	462.6	2.2
M06-2X/6-311++G(df,pd)	7098.9	111.5	486.0	2.3	462.6	2.2
M06-2X/6-311G(d,p)	7096.4	109.0	485.6	1.9	462.3	1.9
M06-2X/6-311+G(d,p)	7084.1	96.7	485.1	1.4	461.7	1.3
M06-2X/6-311++G(d,p)	7083.9	96.5	485.1	1.4	461.7	1.3
M06-2X/6-31G(d,p)	7073.7	86.3	485.6	1.9	462.2	1.8
M06-2X/6-31+G(d,p)	7055.4	68.0	484.2	0.5	460.9	0.5
M06-2X/6-31++G(d,p)	7055.1	67.7	484.2	0.5	460.9	0.5
M06-2X-aug-cc-pVDZ	7060.6	73.2	485.1	1.4	461.8	1.4
M06-2X-aug-cc-pVTZ	7109.6	122.2	486.7	3.0	463.3	2.9
M06-2X-cc-pVDZ	7071.0	83.6	485.9	2.2	462.5	2.1
M06-2X-cc-pVTZ	7111.4	124.0	486.9	3.2	463.5	3.1
MP2/6-311G(3df,3pd)	7060.1	72.7	488.4	4.7	464.6	4.2
MP2/6-311G+(3df,3pd)	7048.6	61.2	487.5	3.8	463.8	3.4
MP2/6-311G++(3df,3pd)	7048.6	61.2	487.5	3.8	463.8	3.4
MP2/6-311G(2d,2p)	7051.6	64.2	486.3	2.6	462.6	2.2
MP2/ 6-311+G(2d,2p)	7033.2	45.8	485.4	1.7	461.7	1.3
MP2/6-311++G(2d,2p)	7032.6	45.2	485.4	1.7	461.8	1.4
MP2/6-311G(2df,2pd)	7069.3	81.9	488.8	5.1	465.0	4.6
MP2/6-311+G(2df,2pd)	7051.0	63.6	487.9	4.2	464.1	3.7
MP2/6-311++G(2df,2pd)	7050.4	63.0	487.9	4.2	464.1	3.7
MP2/6-311G(df,pd)	7060.3	72.9	487.5	3.8	463.8	3.4
MP2/6-311+G(df,pd)	7045.0	57.6	486.5	2.8	462.9	2.5
MP2/6-311++G(df,pd)	7043.7	56.3	486.6	2.9	462.9	2.5
MP2/6-311G(d,p)	7008.4	21.0	484.1	0.4	460.6	0.2
MP2/6-311+G(d,p)	6993.0	5.6	483.4	–0.3	459.8	–0.6
MP2/6-311++G(d,p)	***6990.7***	***3.3***	***483.4***	***–0.3***	***459.9***	***–0.5***
MP2/6-31G(d,p)	6988.2	0.8	484.7	1.0	461.0	0.6
MP2/6-31+G(d,p)	6959.2	–28.2	483.3	–0.4	459.6	–0.8
MP2/6-31++G(d,p)	6954.4	–33.0	483.3	–0.4	459.5	–0.9
MP2/aug-cc-pVDZ	6913.6	–73.8	480.7	–3.0	457.2	–3.2
MP2/cc-pVDZ	6949.3	–38.1	482.4	–1.3	458.9	–1.5
MP2/cc-pVTZ	7039.5	52.1	487.5	3.8	463.7	3.3
ωB97X-D/6-311G(d,p)	7051.5	64.1	483.2	–0.5	459.9	–0.5
ωB97X-D/6-311+G(d,p)	7051.3	63.9	483.2	–0.5	459.9	–0.5
ωB97X-D/6-311++G(d,p)	7065.0	77.6	483.8	0.1	460.5	0.1
ωB97X-D/aug-cc-pVDZ	7021.2	33.8	482.7	–1.0	459.4	–1.0
ωB97X-D/aug-cc-pVTZ	7075.9	88.5	484.9	1.2	461.5	1.1
ωB97X-D/cc-pVDZ	7030.7	43.3	483.3	–0.4	460.0	–0.4
ωB97X-D/cc-pVTZ	7078.8	91.4	485.2	1.5	461.7	1.3
**Expt.**	**3599.0**		**625.4**		**585.8**	

Table S-4. Observed 100 A and 80 E species frequencies ($\nu_{Obs.}$) of conformer I of MHO. $\nu_{Obs.} - \nu_{Calc.}$ values as obtained with the program *XIAM*.

J	K_a	K_c	*J*	K_a	K_c		$\nu_{Obs.}$	$\nu_{Obs.} - \nu_{Calc.}$
upper level			lower level				GHz	kHz
8	0	8	7	0	7	A	9656.6451	–1.9
8	0	8	7	0	7	E	9656.6180	2.3
8	1	7	7	1	6	A	9839.2242	–2.3
8	1	7	7	1	6	E	9839.1749	2.0
8	2	6	7	2	5	A	9717.2734	–2.6
8	2	7	7	2	6	A	9684.7185	–2.6
9	0	9	8	0	8	A	10854.0067	–2.7
9	0	9	8	0	8	E	10853.9786	2.9
9	1	8	8	1	7	A	11066.2934	–2.6
9	1	8	8	1	7	E	11066.2397	1.8
9	1	9	8	1	8	A	10711.7442	–0.8
9	1	9	8	1	8	E	10711.7294	1.5
9	2	7	8	2	6	A	10939.7688	–2.4
9	2	7	8	2	6	E	10939.3282	2.8
9	2	8	8	2	7	A	10893.5377	–3.3
9	2	8	8	2	7	E	10893.9038	0.2
10	0	10	9	0	9	A	12048.1819	–2.3
10	0	10	9	0	9	E	12048.1521	3.5
10	1	9	9	1	8	A	12292.2924	–3.1
10	1	9	9	1	8	E	12292.2346	2.1
10	1	10	9	1	9	A	11899.0506	–0.1
10	1	10	9	1	9	E	11899.0328	1.9
10	2	8	9	2	7	A	12164.7854	–2.6
10	2	8	9	2	7	E	12164.5020	2.8
10	2	9	9	2	8	A	12101.7351	–3.3
10	2	9	9	2	8	E	12101.9349	1.1
10	3	8	9	3	7	A	12119.9612	–3.9
10	3	8	9	3	7	E	12120.7803	1.4
1	1	1	0	0	0	A	4184.9260	–1.0
1	1	1	0	0	0	E	4184.2045	–1.2
1	1	0	1	0	1	A	3013.2520	–11.3
1	1	0	1	0	1	E	3013.7037	–2.9
2	2	0	1	1	1	A	11422.5699	1.2
2	2	0	1	1	1	E	11432.1877	–3.3
2	2	1	1	1	0	A	11382.6439	–2.0
2	2	1	1	1	0	E	11372.1825	–1.2
2	1	1	2	0	2	A	3053.2148	10.2
2	1	1	2	0	2	E	3053.2582	–1.3
2	2	1	2	1	2	A	9039.3035	0.4
2	2	0	2	1	2	A	9039.6940	–0.7
2	2	0	2	1	2	E	9048.9341	–2.5

2	2	0	2	1	1	A	8921.1008	–1.3
2	2	0	2	1	1	E	8929.9740	11.9
3	1	3	2	0	2	A	6508.6694	–0.6
3	1	3	2	0	2	E	6508.4321	–1.5
3	2	1	2	1	2	A	12674.9394	–3.6
3	2	1	2	1	2	E	12683.4258	–9.2
3	2	2	2	1	1	A	12554.3920	–0.7
3	2	2	2	1	1	E	12545.0422	–9.5
3	3	0	2	2	1	A	18598.9372	–0.6
3	3	1	2	2	0	A	18598.5501	6.3
3	2	2	3	1	3	A	9098.9254	–2.0
3	2	2	3	1	3	E	9089.8709	–6.6
3	2	1	3	1	3	A	9100.8827	–2.3
3	2	1	3	1	3	E	9109.2811	–5.2
3	2	1	3	1	2	A	8863.7064	1.4
3	2	1	3	1	2	E	8871.9317	0.0
3	3	1	3	2	2	A	14965.2499	–3.2
3	3	1	3	2	2	E	14958.8231	–1.5
3	3	0	3	2	2	A	14965.2499	–5.6
3	3	0	3	2	1	A	14963.3018	3.9
3	3	0	3	2	1	E	14968.3592	2.0
4	1	4	3	0	3	A	7641.6074	–2.4
4	1	4	3	0	3	E	7641.4181	1.5
4	2	2	3	1	3	A	13949.3751	–8.1
4	2	2	3	1	3	E	13956.1974	2.5
4	2	3	3	1	2	A	13706.3299	–2.5
4	2	3	3	1	2	E	13698.6623	0.2
4	2	3	4	1	4	A	9178.5725	1.8
4	2	3	4	1	4	E	9171.0394	–6.0
4	2	2	4	1	4	A	9184.4391	–2.4
4	2	2	4	1	4	E	9191.2192	–4.4
5	1	5	4	0	4	A	8756.2152	–1.0
5	1	5	4	0	4	E	8756.0486	0.1
5	2	3	4	1	4	A	15247.4663	–3.5
5	2	3	4	1	4	E	15252.0692	8.4
5	2	4	4	1	3	A	14838.5000	0.6
5	2	4	4	1	3	E	14833.0378	–1.8
5	2	4	5	1	5	A	9278.3515	2.1
5	2	4	5	1	5	E	9272.9601	–2.9
5	2	3	5	1	5	A	9292.0412	2.4
5	2	3	5	1	5	E	9296.6221	–0.2
5	2	3	5	1	4	A	8699.1702	–1.5
5	2	3	5	1	4	E	8703.7324	0.4
5	3	3	5	2	4	A	14968.9518	–3.2
5	3	3	5	2	4	E	14958.8231	1.1
5	3	2	5	2	4	A	14969.0230	0.1
6	1	6	5	0	5	A	9853.5771	–3.3

6	1	6	5	0	5	E	9853.4323	0.9
6	2	4	5	1	5	A	16571.1871	2.5
6	2	4	5	1	5	E	16573.8163	3.6
6	3	3	5	2	4	A	22238.7905	10.6
6	2	5	6	1	6	A	9398.4001	0.1
6	2	5	6	1	6	E	9394.9157	–2.5
6	2	4	6	1	6	A	9425.7504	1.8
6	2	4	6	1	6	E	9428.3810	1.2
6	2	4	6	1	5	A	8595.8650	2.1
6	2	4	6	1	5	E	8598.5163	0.3
6	3	3	6	2	5	A	14973.3004	7.0
6	3	3	6	2	4	A	14945.9508	6.1
6	3	3	6	2	4	E	14956.5684	0.4
6	4	3	6	3	4	A	20946.2859	–6.2
6	4	2	6	3	4	A	20946.2859	–6.8
7	3	4	7	2	6	A	14980.0850	4.1
8	1	8	7	0	7	A	12002.4189	–4.4
8	1	8	7	0	7	E	12002.3096	4.6
8	3	6	8	2	7	A	14989.1209	–12.3
8	3	6	8	2	7	E	14975.6545	–0.3
8	3	5	8	2	6	A	14908.5553	1.0
8	3	5	8	2	6	E	14920.6681	–0.5
9	1	9	8	0	8	A	13057.5214	0.2
9	1	9	8	0	8	E	13057.4205	3.3
9	3	6	9	2	8	A	15004.8229	1.6
9	3	6	9	2	8	E	15018.2241	–0.4
9	3	6	9	2	7	A	14876.8936	0.2
10	1	10	9	0	9	A	14102.5587	–3.8
10	1	10	9	0	9	E	14102.4754	3.1
10	3	7	10	2	9	A	15024.9603	0.1
10	3	7	10	2	9	E	15037.2655	1.0
1	1	0	0	0	0	A	4224.4583	0.1
1	1	0	0	0	0	E	4224.8959	–1.0
2	1	1	1	0	1	A	5475.2001	1.4
2	1	1	1	0	1	E	5475.2428	–1.5
2	2	1	1	1	1	A	11422.1727	–4.4
2	2	1	1	1	1	E	11412.8867	11.8
2	2	0	1	1	0	A	11383.0386	1.1
2	2	0	1	1	0	E	11391.4993	–0.5
2	2	1	2	1	1	A	8920.7068	–3.7
2	2	1	2	1	1	E	8910.6402	–5.7
3	1	2	2	0	2	A	6745.8465	–3.5
3	1	2	2	0	2	E	6745.7888	0.6
3	2	2	2	1	2	A	12672.9917	6.3
3	2	1	2	1	1	A	12556.3500	–0.4
3	2	1	2	1	1	E	12564.4557	–4.8
3	3	1	2	2	1	A	18598.9370	1.7

3	3	1	2	2	1	E	18593.2345	4.2
3	3	0	2	2	0	A	18598.5501	3.9
3	3	0	2	2	0	E	18602.8554	–0.2
3	2	2	3	1	2	A	8861.7479	0.5
3	2	2	3	1	2	E	8852.5325	9.6
3	3	1	3	2	1	A	14963.3018	6.3
4	1	3	3	0	3	A	8036.8856	–5.0
4	1	3	3	0	3	E	8036.7813	2.7
4	2	3	3	1	3	A	13943.5147	2.3
4	2	3	3	1	3	E	13936.0125	–4.2
4	2	2	3	1	2	A	13712.2033	0.1
4	2	2	3	1	2	E	13718.8381	–2.2
4	3	2	3	2	2	A	19811.1100	0.0
4	3	2	3	2	2	E	19804.6768	4.3
4	3	1	3	2	1	A	19809.1740	4.7
4	3	1	3	2	1	E	19814.2021	–0.4
5	1	4	4	0	4	A	9349.0822	–1.1
5	1	4	4	0	4	E	9348.9405	1.8
5	2	4	4	1	4	A	15233.7794	–0.9
5	2	3	4	1	3	A	14852.1876	–1.3
5	3	3	4	2	3	A	21024.1687	4.2
5	3	3	4	2	3	E	21016.1813	3.1
5	3	2	4	2	2	A	21018.3628	1.2
5	3	2	4	2	2	E	21024.9348	–0.3
5	2	4	5	1	4	A	8685.4811	–1.1
5	2	4	5	1	4	E	8680.0719	–0.9
5	3	3	5	2	3	A	14955.2693	3.8
6	1	5	5	0	5	A	10683.4638	–2.3
6	1	5	5	0	5	E	10683.2963	1.1
6	2	5	5	1	5	A	16543.8352	–0.7
6	2	5	5	1	5	E	16540.3528	1.7
6	3	4	5	2	4	A	22238.5642	–12.1
6	3	4	5	2	4	E	22228.4905	3.4
6	2	5	6	1	5	A	8568.5138	–0.5
6	2	5	6	1	5	E	8565.0541	–0.3
6	3	4	6	2	4	A	14945.7369	–4.2
6	3	4	6	2	4	E	14927.6380	0.6
7	1	6	6	0	6	A	12041.3361	–1.0
7	1	6	6	0	6	E	12041.1429	1.3
8	1	7	7	0	7	A	13424.2261	–4.2
8	1	7	7	0	7	E	13424.0128	3.0
8	3	6	8	2	6	A	14907.4368	1.3
9	1	8	8	0	8	A	14833.8796	0.3
9	1	8	8	0	8	E	14833.6366	4.6
10	3	8	10	2	8	A	14829.8353	0.7

Table S-5a. Molecular constants in the rho axis system of conformer C_1 of MHO obtained by a fit using the program *BELGI-C₁*.

Operator[a]	Constant[b]	Units	Values[c]
$\mathbf{P_a^2}$	A	MHz	3507.798(86)
$\mathbf{P_b^2}$	B	MHz	715.01(23)
$\mathbf{P_c^2}$	C	MHz	587.42(32)
$\{\mathbf{P_a}, \mathbf{P_b}\}$	D_{ab}	GHz	–0.53[d]
$-\mathbf{P^4}$	Δ_J	kHz	0.33723(76)
$-\mathbf{P^2 P_a^2}$	Δ_{JK}	kHz	–10.195(10)
$-\mathbf{P_a^4}$	Δ_K	kHz	53.514(27)
$-2\mathbf{P^2}(\mathbf{P_b^2} - \mathbf{P_c^2})$	δ_J	kHz	0.09894(50)
$-\{\mathbf{P_a^2}, (\mathbf{P_b^2} - \mathbf{P_c^2})\}^2$	δ_K	kHz	–1.012(31)
$\mathbf{P_\alpha^2}$	F	cm^{-1}	5.268[e]
$(1/2)[1 - \cos(3\alpha)]$	V_3	cm^{-1}	408.35(11)
$\mathbf{P_a P_\alpha}$	ρ	unitless	0.016138(23)
$\{\mathbf{P_a}, \mathbf{P_c}\}$	D_{acI}	GHz	0.0378(37)
	N_A/N_E		100/80
	rms	kHz	4.3/5.1

[a] All constants refer to a rho-axis system, therefore the inertia tensor is not diagonal and the constants cannot be directly compared to those of a principal axis system given in Table 2. $\mathbf{P_a}$, $\mathbf{P_b}$, $\mathbf{P_c}$ are the components of the overall rotation angular momentum, $\mathbf{P_\alpha}$ is the angular momentum of the internal rotor rotating around the internal rotor axis by an angle α. {u,v} is the anti commutator uv + vu.

[b] The product of the parameter and operator from a given row yields the term actually used in the rotation-torsion Hamiltonian, except for F, ρ, and A, which occur in the Hamiltonian in the form $F(\mathbf{P_\alpha} - \rho\mathbf{P_a^2}) + A\mathbf{P_a^2}$.

[c] Values of the parameters from the present fit. Statistical uncertainties are shown as one standard uncertainty in the last digit.

[d] Fixed to the value from a previous fit.

[e] Fixed to the *XIAM* value.

Table S-5b. Molecular constants in the rho axis system of conformer C_s of MHO obtained by a fit using the program *BELGI-C_s*.

Operator[a]	Constant[b]	Units	Values[c]
$\mathbf{P_a^2}$	A	MHz	6809(14)
$\mathbf{P_b^2}$	B	MHz	511.0(21)
$\mathbf{P_c^2}$	C	MHz	472.9(18)
$\{\mathbf{P_a}, \mathbf{P_b}\}$	D_{ab}	GHz	–0.48[d]
$\mathbf{P_\alpha^2}$	F	cm^{-1}	5.268[e]
$(1/2)[1-\cos(3\alpha)]$	V_3	cm^{-1}	395.5(33)
$\mathbf{P_a P_\alpha}$	ρ	unitless	0.0322(13)
$[1-\cos(3\alpha)]\mathbf{P_a^2}$	k_5	MHz	4.086(42)
$[1-\cos(3\alpha)]\mathbf{P^2}$	F_v	MHz	–12.08(22)
$\mathbf{P_a P_\alpha P^2}$	L_v	kHz	–5.9(18)
$[1-\cos(3\alpha)](\mathbf{P_b^2}-\mathbf{P_c^2})$	c_2	MHz	29.1(63)
$\mathbf{P_\alpha^2}(\mathbf{P_b^2}-\mathbf{P_c^2})$	c_1	MHz	0.447(69)
	N_A/N_E		21/21

[a] All constants refer to a rho-axis system, therefore the inertia tensor is not diagonal and the constants cannot be directly compared to those of a principal axis system given in Table 2. $\mathbf{P_a}$, $\mathbf{P_b}$, $\mathbf{P_c}$ are the components of the overall rotation angular momentum, $\mathbf{P_\alpha}$ is the angular momentum of the internal rotor rotating around the internal rotor axis by an angle α. {u,v} is the anti commutator uv + vu.

[b] The product of the parameter and operator from a given row yields the term actually used in the rotation-torsion Hamiltonian, except for F, ρ, and A, which occur in the Hamiltonian in the form $F(\mathbf{P_\alpha} - \rho\mathbf{P_a^2}) + A\mathbf{P_a^2}$.

[c] Values of the parameters from the present fit. Statistical uncertainties are shown as one standard uncertainty in the last digit.

[d] Fixed to the *ab initio* value.

[e] Fixed to the *XIAM* value.

Table S-6. Observed 21 A and 21 E species frequencies ($\nu_{Obs.}$) of conformer XIII of MHO. $\nu_{Obs.} - \nu_{Calc.}$ values as obtained with the program *XIAM*.

J	***K*a**	***K*c**	***J***	***K*a**	***K*c**		**$\nu_{Obs.}$**	**$\nu_{Obs.} - \nu_{Calc.}$**
upper level			lower level				GHz	kHz
2	1	2	1	0	1	A	8369.3315	–4.7
2	1	2	1	0	1	E	8365.3963	–10.3
3	1	3	2	0	2	A	9278.6122	–0.6
3	1	3	2	0	2	E	9276.0759	7.9
4	1	4	3	0	3	A	10176.4023	0.3
4	1	4	3	0	3	E	10174.4447	4.4
5	1	5	4	0	4	A	11062.8250	–2.7
5	1	5	4	0	4	E	11061.1631	1.1
6	1	6	5	0	5	A	11938.0615	0.8
6	1	6	5	0	5	E	11936.5696	3.7
7	1	7	6	0	6	A	12802.3215	2.7
7	1	7	6	0	6	E	12800.9365	4.3
8	1	8	7	0	7	A	13655.8640	–3.0
8	1	8	7	0	7	E	13654.5511	–2.8
9	1	9	8	0	8	A	14499.0170	–0.3
9	1	9	8	0	8	E	14497.7560	–0.9
10	1	10	9	0	9	A	15332.1275	–1.2
10	1	10	9	0	9	E	15330.9106	2.5
11	1	11	10	0	10	A	16155.6055	–1.2
11	1	11	10	0	10	E	16154.4169	–0.6
12	1	12	11	0	11	A	16969.9042	1.4
12	1	12	11	0	11	E	16968.7378	–1.7
13	1	13	12	0	12	A	17775.5145	0.7
13	1	13	12	0	12	E	17774.3726	0.1
14	1	14	13	0	13	A	18572.9758	–4.1
14	1	14	13	0	13	E	18571.8593	1.2
15	1	15	14	0	14	A	19362.8827	–0.6
15	1	15	14	0	14	E	19361.7827	3.4
16	1	16	15	0	15	A	20145.8441	–1.4
16	1	16	15	0	15	E	20144.7595	1.2
4	1	3	4	0	4	A	6632.6961	–2.1
4	1	3	4	0	4	E	6632.5107	2.6
4	2	3	4	1	4	A	19664.1047	5.5
5	2	4	5	1	5	A	19722.1582	–9.2
5	2	4	5	1	5	E	19691.9458	–6.6
6	2	5	6	1	6	A	19791.9125	5.9
6	2	5	6	1	6	E	19762.5366	3.6
7	2	6	7	1	7	A	19873.3488	1.2
2	2	0	1	1	0	E	21443.1443	4.6
3	2	1	2	1	1	E	22368.1196	–3.1
4	2	2	3	1	2	E	23279.0544	–4.6
5	2	3	5	1	4	A	19376.7035	2.8
